# Different Multivariable Risk Factors for Rapid Progressive Interstitial Lung Disease in Anti-MDA5 Positive Dermatomyositis and Anti-Synthetase Syndrome

**DOI:** 10.3389/fimmu.2022.845988

**Published:** 2022-03-07

**Authors:** Yu Zuo, Lifang Ye, Fang Chen, Yawen Shen, Xin Lu, Guochun Wang, Xiaoming Shu

**Affiliations:** ^1^ Department of Rheumatology, Key Laboratory of Myositis, China-Japan Friendship Hospital, Beijing, China; ^2^ Peking Union Medical College, Chinese Academy of Medical Sciences, Beijing, China; ^3^ Department of Rheumatology, Beijing Shijitan Hospital, Capital Medical University, Beijing, China

**Keywords:** idiopathic inflammatory myopathy, anti-MDA5 antibody, anti-synthetase syndrome, rapidly progressive interstitial lung disease, hyperferritinemia

## Abstract

**Background:**

Interstitial lung disease (ILD) is frequently observed in anti-melanoma differentiation-associated protein 5 (MDA5) antibody positive dermatomyositis (DM) and anti-synthetase syndrome (ASS), where they often develop a rapidly progressive ILD (RP-ILD) leading to poor prognosis.

**Objective:**

The aim of this study was to construct multivariable prediction risk factors for rapid progressive ILD (RP-ILD) in anti-MDA5 positive DM (MDA5^+^DM) and ASS.

**Methods:**

333 idiopathic inflammatory myopathy (IIM) associated ILD patients were studied retrospectively. Risk factors for RP-ILD in MDA5^+^DM and ASS patients were identified by univariate and multivariable logistic regression analysis. The mortality was assessed using Kaplan-Meier analysis.

**Results:**

RP-ILD was more prevalent in MDA5^+^DM patients than ASS patients. MDA5^+^DM patients with RP-ILD had significantly lower survival rates than those in ASS patients. The independent risk factors for RP-ILD in MDA5^+^DM patients were fever (OR 3.67, 95% CI:1.79-7.52), lymphopenia (OR 2.14, 95% CI:1.01-4.53), especially decreased levels of CD3^+^T cells (OR 2.56, 95% CI:1.17-5.61), decreased levels of CD3^+^CD4^+^ T cells (OR 2.80, 95% CI:1.37-5.73), CD3^+^CD8^+^T cells (OR 2.18, 95% CI:1.05-4.50), elevated CD5^-^CD19^+^ B cells (OR 3.17, 95% CI:1.41-7.13), elevated ALT (OR 2.36, 95% CI:1.15-4.81), high lactate dehydrogenase (LDH) (OR 3.08, 95% CI:1.52-6.27), hyper-ferritin (OR 4.97, 95% CI:1.97-12.50), elevated CEA (OR 2.28, 95% CI:1.13-4.59), and elevated CA153 (OR 3.31, 95% CI:1.50-7.27). While the independent risk factors for RP-ILD in ASS patients were elevated CEA (OR 5.25, 95% CI: 1.73-15.93), CA125 (OR 2.79, 95% CI: 1.10-7.11) and NSE (OR 4.86, 95% CI: 1.44-16.37). Importantly, serum ferritin>2200ng/ml predicted patient’s death within half a year in MDA5^+^DM patients with RP-ILD, but not in ASS patients.

**Conclusions:**

There were significant different mortality and multivariable risk factors for RP-ILD in MDA5^+^DM patients and ASS patients. Potential clinical benefits of using these different risk factors deserve assessment of severity and prognosis in IIM patients.

## Introduction

IIM comprises a group of systemic autoimmune disorders, including DM, polymyositis, ASS, immune-mediated necrotizing myopathy (IMNM), inclusion body myositis (IBM), affecting skeletal muscles and other organs. In patients with IIM, interstitial lung disease (ILD) is a common extramuscular involvement associated with poor prognosis (1). RP-ILD is defined as a progressive deterioration associated with ILD within 3 months (2).

In the past year, there have been several studies that reported on the risk factor model of amyopathic dermatomyositis combined with ILD, which provides a favorable basis for better clinical assessment of disease progression (3, 4). However, these studies did not separately discuss risk factors and prognosis of RP-ILD in MDA5^+^DM and ASS patients.

Researches in recent years have shown that myositis-specific antibody (MSA) profiles help IIM classification. Different antibody-positive IIM has different clinical characteristics. Our previous research showed that ILD had different types in patients with different antibodies (5), in addition, RP-ILD was found both in MDA5^+^DM and ASS patients. Our previous study has reported that anti-MDA5 was an independent risk factor for RP-ILD (6). ASS patients with ILD generally have slower disease progression, however, another research from our study showed that anti-threonyl tRNA synthetase (PL-7), one of the subtypes of anti-aminoacyl-tRNA synthetase (ARS) antibodies, was closely associated with RP-ILD (7). However, there is no report of exact clinical difference between RP-ILD patients with anti-MDA5 and anti-ARS. Additionally, it is still unclear as to the disparity in survival between the two groups. Furthermore, hyperferritinemia has been proven to be the hallmark and a predictor of poor outcome of ILD associated with MDA5^+^DM, however different studies have different cut-off values of ferritin for prognosis (8, 9) and as far as we know, there is no separate study on ferritin in the RP-ILD population.

Thus, this retrospective study was conducted to elucidate clinical difference and survival rates of RP-ILD in anti-MDA5 positive and anti-ARS positive patients, to analyze the susceptibility factors of RP-ILD in two groups of patients separately. Furthermore, prognostic value of serum ferritin in anti-MDA5 positive patients with RP-ILD was evaluated.

## Materials and Methods

### Patient Population

A total of 333 patients with IIM associated ILD (IIM-ILD) were enrolled in this study. 175 patients were positive for anti-MDA5 antibody, and 158 patients were positive for anti-ARS antibodies. All patients were treated in China-Japan Friendship Hospital between July 2013 and October 2018. DM was diagnosed on the basis of the Bohan and Peter criteria, and 239th European Neuro Muscular Centre International Workshop guidelines (10, 11). ASS was diagnosed with definitive serology findings of one of anti-ARS antibodies tested, along with at least one triad finding, including myositis, arthritis, and ILD (12). The exclusion criteria were patients with infections, cancers and/or other CTDs. All patient data were used anonymously with written informed consent from all participants. This study was approved by the Human Ethics Board of the China-Japan Friendship Hospital (approval number: 2016-117).

### Data Collection

Clinical data collected included demographics, clinical characteristics, laboratory findings containing auto-antibodies, creatine kinase (CK), serum ferritin, and tumor marker levels; pulmonary function test results (forced vital capacity (FVC), forced expiratory volume in the first second (FEV1) and diffusing capacity of the lung for carbon monoxide; BALF analyses mainly included cell types of fluid. All these routine tests were done in all patients at the first visit in our cohort. Among 333 patients, 122 patients had lung function tests. 100 patients had BALF analyses, and mainly performed when ILD worsened. The follow-up time was defined as the time between the first visit for our cohort to the death or the end of 28 February 2019.

ILD was divided into two categories based on clinical manifestations: RP-ILD and chronic ILD. RP-ILD was defined as displaying two or more of the following within 3 months: 1) dyspnea exacerbation; 2) an increase in parenchymal abnormality on high-resolution computed tomography (HRCT) scan; and 3) one of the following physiological changes: >10% decrease in vital capacity (VC) or >1.33 kPa decrease in arterial oxygen tension (PaO_2_). The chronic form was defined as a slowly progressive ILD with gradual deterioration over 3 months (2).

### Autoantibody Analyses

The autoantibody tests were performed on the patients’ first admission. The serum anti-nuclei antibody profiles and MSAs including anti-MDA5, anti-histidyl tRNA synthetase (Jo-1), anti-threonyl tRNA synthetase (PL-7), anti-alanyl tRNA synthetase (PL-12), anti-isoleucyl-tRNA synthetase (OJ), and anti-glycyl-tRNA synthetase (EJ) were tested using commercially available kits (EUROIMMUN, Lübeck, Germany) according to the manufacturer’s instructions.

### Analysis of Lymphocyte Subsets in Peripheral Blood of Patients With DM

Lymphocyte subsets analyses were underwent in 286 patients at the first visit in our cohort. A part of patients (n=92) did not receive glucocorticoids therapy before, however, there were still a number of patients (n=194) received glucocorticoids therapy for a short time at other hospitals, which may affect the predictive value of lymphocyte subsets. The counts of CD3^+^CD4^+^T cells, CD3^+^CD8^+^T cells, CD3^-^CD19^+^B cells and CD3^-^CD16^+^CD56^+^ NK cells in peripheral blood were detected by flow cytometry (Beckman Coulter, USA) using specific monoclonal antibodies (Beckman Coulter, USA), respectively. Data were analyzed using Cytomics FC500 (Beckman Coulter, USA).

### Statistical Analysis

Statistical analysis was performed using SPSS version 11.0 (SPSS Inc. Chicago, IL, U.S.A.). For group comparisons involving demographic characteristics, clinical symptoms and laboratory tests, we used χ^2^ tests for the categorical data and the Mann–Whitney U test or two-sample t-tests to analyze continuous data. Univariate and multivariate logistic regression analysis were used to determine the predictors of RP-ILD. The prediction was quantified by the odds ratio with its 95% CI and P<0.05 was considered statistically significant. Survival curves were drawn by the Kaplan-Meier method. The log-rank test was used to compare survival rates. In order to compare the predictive performance of serum ferritin, ROC analysis was performed. We calculated alternative cut-off point by the Youden’s index.

## Results

### Clinical Features and Prevalence of RP-ILD of MDA5^+^DM and ASS Patients

The demographics, clinical manifestations, laboratory test results at diagnosis, pulmonary function test results, and BALF analyses of the 333 enrolled patients with IIM-ILD having either anti-MDA5 or anti-ARS antibodies are summarized in **Table 1**. The mean age at disease onset was 49.7 ± 12.3 years. The majority of the patients were women (M: F ratio = 1:2.3). Of the 333 patients, 175 (52.6%) were positive for anti-MDA5 antibody and 158 (47.4%) were positive for anti-ARS (including 79 anti-Jo-1, 35 anti-PL-7, 17 anti-PL-12, 27 anti-EJ respectively) antibodies. We also found that anti-Ro-52 was identified as the most frequent myositis-associated autoantibody in 113 (33.9%) patients.

**Table 1:** Characteristics of study participants at diagnosis.

	Values	Range
Age on set, years	49.7 ± 12.3	(7-79)
Male/Female	102/231 (1:2.3)
Fever	158 (47.4%)
CK, IU/L (Normal 26-200)	433.9 ± 1082.8	(12-10762)
Ferritin, ng/mL (Normal 11-306.8)	793.0 ± 1370.0	(5.7-11871)
Anti-MDA5 antibody	175 (52.6%)
Anti-ARS antibodies	158 (47.4%)
Anit-Jo-1 antibody	79 (23.7)
Anti-PL-7 antibody	35 (10.5)
Anti-PL-12 antibody	17 (5.1)
Anti-EJ antibody	27 (8.1)
Anti-Ro-52 antibody	113 (33.9%)
FVC%	74.8 ± 19.2	(39-140)
FEV1%	71.8 ± 16.9	(40-112)
DLCO%	54.9 ± 15.5	(29-100)
BALF LYM%	24.4 ± 23.1	(0-97)
BALF NE%	50.7 ± 28.2	(2-99)
BALF Mac%	20.8 ± 15.8	(1-74)
Treatment of MDA5^+^DM (n, %)		
Glucocorticoids	175 (100%)	
Pulse steroid therapy	8 (4.6%)	
Calcineurin inhibitors	70 (40.0%)	
Cyclophosphamide	42 (24.0%)	
Mycophenolate mofetil	10 (5.7%)	
Treatment of ASS (n, %)		
Glucocorticoids	158 (100%)	
Pulse steroid therapy	2 (1.3%)	
Calcineurin inhibitors	12 (7.6%)	
Cyclophosphamide	64 (40.5%)	
Mycophenolate mofetil	17 (10.8%)	

FVC%, percentage of the predicted forced vital capacity; FEV1%, percent of forced expiratory volume in the first second; DLCO%, percentage of the predicted diffusion capacity for carbon monoxide; BALF LYM%, percentage of lymphocyte in bronchoalveolar lavage fluid; BALF NE%, percentage of neutrophil in bronchoalveolar lavage fluid; BALF Mac%, percentage of macrophage in bronchoalveolar lavage fluid.

Among the 333 patients with IIM-ILD, 180 (54.1%) experienced a RP pattern, of whom 110 (61.1%) were anti-MDA5 antibody-positive and 70 (38.9%) were anti-ARS antibody-positive. The percentage of RP-ILD was significantly higher in MDA5^+^DM patients than in ASS patients (62.9% *vs.* 44.3%; *p* = 0.001; **Table 2**). The frequencies of RP-ILD varied among the anti-ARS subgroups; however, no statistically significant differences were observed.

**Table 2:** Frequencies of RP-ILD among patients with different MSAs.

MSAs	Cases	RP-ILD	Chronic ILD	*P* value*
Total	333	180(54.1)	153(45.9)	
Anti-MDA5	175	110(62.9)	65(37.1)	0.001
Anti-ARS	158	70(44.3)	88(55.7)	0.001
Jo-1	79	34(43.0)	45(57.0)	0.024
PL-7	35	19(54.3)	16(45.7)	0.977
PL-12	17	6(35.3)	11(64.7)	0.111
EJ	27	11(40.7)	16(59.3)	0.148

MSAs, myositis specific autoantibodies. *indicated that the results were certain MSA positive group comparison with negative group.

### Variables Associated With RP-ILD in MDA5^+^DM and ASS Patients

In first, we identified the incidence of typical rash, including the heliotrope, Gottron, V, and Shawl signs, and skin ulcer and the levels of serum alanine transaminase (ALT), aspartate transaminase (AST), and ferritin were significantly higher, whereas CK levels in MDA5^+^ DM patients with RP-ILD were much lower (all p values < 0.05). The counts of lymphocytes in the peripheral blood (PBL) were also significantly lower in MDA5^+^DM patients with RP-ILD. Further the counts of CD3^+^CD4^+^ T cells, CD3^+^CD8^+^T cells, CD5^+^CD19^+^ B cells, and NK cells were much lower, whereas the proportion of CD5^-^CD19^+^ B cells was higher in MDA5^+^ DM patients with RP-ILD than in ASS patients (all p values < 0.05) (**Table 3**).

**Table 3:** Clinical difference between anti-MDA5 positive and anti-ARS positive patients with RP-ILD.

Variable	ARS, n=70	MDA5,n=110	*P* value
Heliotrope sign (%)	18.6 (13/70)	73.6 (81/110)	<0.001
Gottron sign (%)	31.4 (22/70)	80.9 (89/110)	<0.001
V sign (%)	14.3 (10/70)	50.9 (56/110)	<0.001
Shawl sign (%)	8.6 (6/70)	34.5 (38/110)	<0.001
Skin ulcer (%)	1.4 (1/70)	24.5 (27/110)	<0.001
CK (IU/L)	541.0 ± 1430.6	250.2 ± 747.3	0.001
ALT (IU/L)	43.4 ± 34.2	87.9 ± 89.7	<0.001
AST (IU/L)	40.3 ± 32.0	111.9 ± 241.8	<0.001
Ferritin (ng/ml)	374.7 ± 461.2	1415.3 ± 1991.1	<0.001
PBL (cell/ul)	1598.0 ± 947.2	770.6 ± 487.4	<0.001
CD3+T	1107.7 ± 655.7	548.9 ± 354.7	<0.001
CD4+T	690.4 ± 546.7	359.4 ± 249.9	<0.001
CD8+T	428.2 ± 357.8	189.2 ± 165.0	<0.001
NK	186.8 ± 189.8	39.6 ± 46.9	<0.001
NK (%)	12.1 ± 7.7	5.5 ± 5.3	<0.001
B1 (CD5+CD19+)	75.4 ± 113.4	23.2 ± 37.2	<0.001
B2 (CD5-CD19+)%	11.9 ± 8.4	19.7 ± 11.7	<0.001
BALF Mac%	14.9 ± 11.5	21.2 ± 15.5	0.090
BALF LYM%	19.3 ± 20.5	27.7 ± 25.0	0.096
BALF NE%	56.7 ± 28.6	47.6 ± 27.3	0.184

BALF Mac%, percentage of macrophage in bronchoalveolar lavage fluid; BALF LYM%, percentage of lymphocyte in bronchoalveolar lavage fluid; BALF NE%, percentage of neutrophil in bronchoalveolar lavage fluid.

### Risk Factors Independently Associated With RP-ILD in MDA5^+^DM and ASS Patients

**Figure 1:**
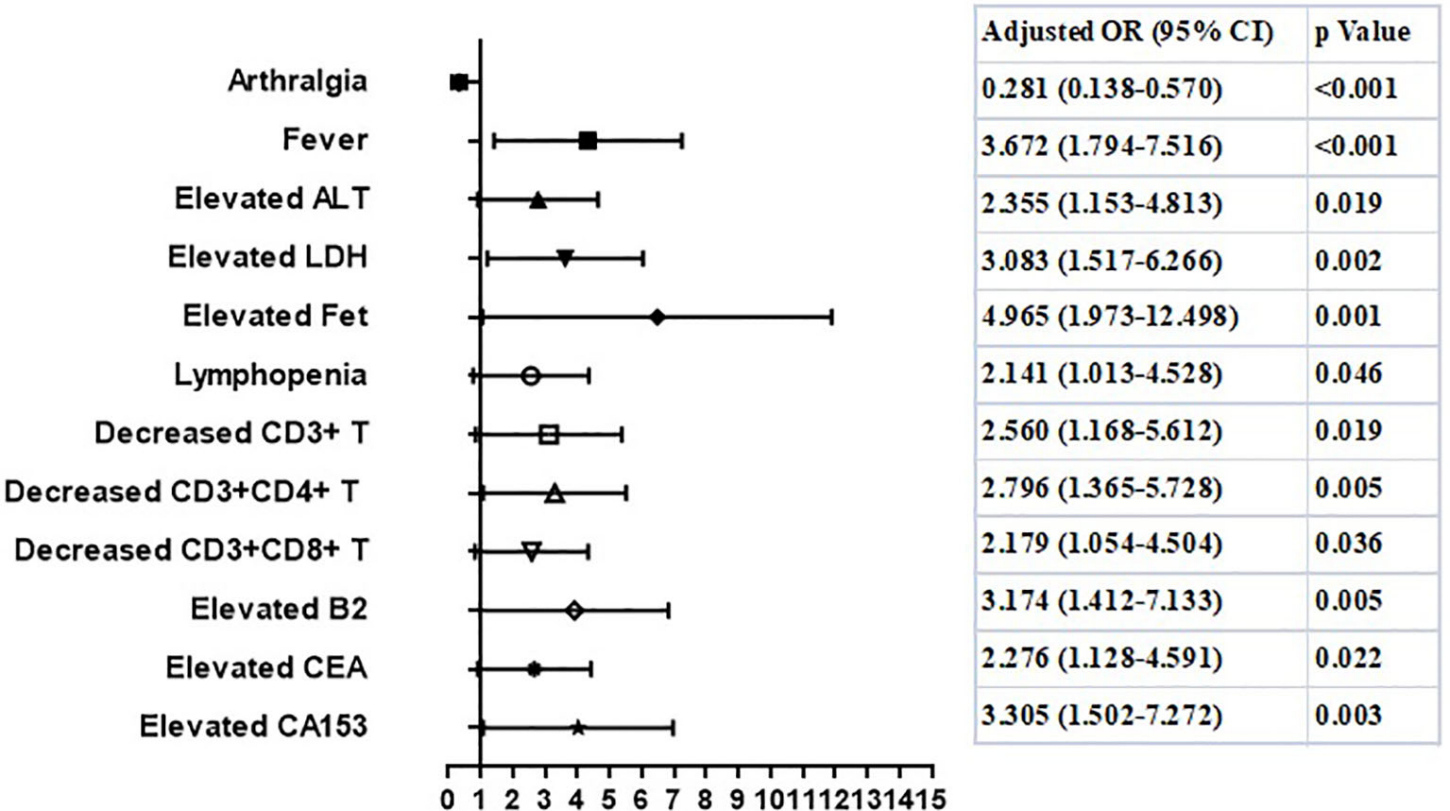
Risk factors of RP-ILD in patients with anti-MDA5 antibody.

To determine the independent risk factors for RP-ILD in either MDA5^+^ DM or ASS patients, we employed univariate and multivariate analyses to identified that fever (OR 3.67, 95% CI:1.79-7.52, p=0.000), lymphopenia (OR 2.14, 95% CI:1.01-4.53, p=0.046), especially decreased levels of CD3^+^T cells (OR 2.56, 95% CI:1.17-5.61, p=0.019), decreased levels of CD3^+^CD4^+^ T cells (OR 2.80, 95% CI:1.37-5.73, p=0.005), CD3^+^CD8^+^T cells (OR 2.18, 95% CI:1.05-4.50, p=0.036), elevated CD5^-^CD19^+^ B cells (OR 3.17, 95% CI:1.41-7.13, p=0.005), elevated ALT (OR 2.36, 95% CI:1.15-4.81, p=0.019), high LDH (OR 3.08, 95% CI:1.52-6.27, p=0.002), hyperferritinemia (OR 4.97, 95% CI:1.97-12.50, p=0.001), elevated CEA (OR 2.28, 95% CI:1.13-4.59, p=0.022), and elevated CA153 (OR 3.31, 95% CI:1.50-7.27, p=0.003) were risk factors for RP-ILD in MDA5^+^DM patients. Interestingly, arthralgia was identified as a protective factor for the development of RP-ILD in MDA5^+^DM patients (**Figure 1**).

**Figure 2:**
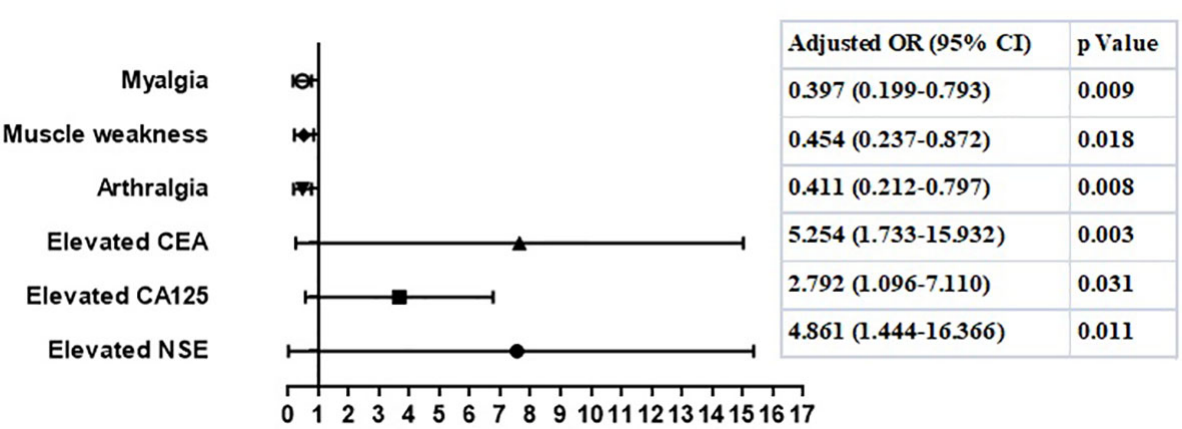
Risk factors of RP-ILD in patients with anti-ARS antibody.

In deviation with MDA5^+^DM patients, the age and sex adjusted multivariate analyses showed that fever, increased levels of serum ferritin, ALT, or LDH, lymphocytes subpopulations including CD3^+^T cells, CD3^+^CD4^+^ T cells, CD3^+^CD8^+^T cells were not risk factors for RP-ILD in ASS patients. Of note, elevated tumor marker CEA (OR 5.25, 95% CI: 1.73-15.93, p=0.003), CA125 (OR 2.79, 95% CI: 1.10-7.11, p=0.031) and NSE (OR 4.86, 95% CI: 1.44-16.37, p=0.011) were both significant independent risk factors for RP-ILD in ASS patients (**Figure 2**). Further, the subtypes of anti-ARS antibodies including anti-Jo-1, anti-PL-7, anti-PL-12, and anti-EJ, were not related to high risk for RP-ILD. Similar to that in MDA5^+^DM, arthralgia, myalgia, and muscle weakness appeared to be protective factors for RP-ILD in ASS patients.

### Mortality in RP-ILD in MDA5^+^DM and ASS Patients

**Figure 3:**
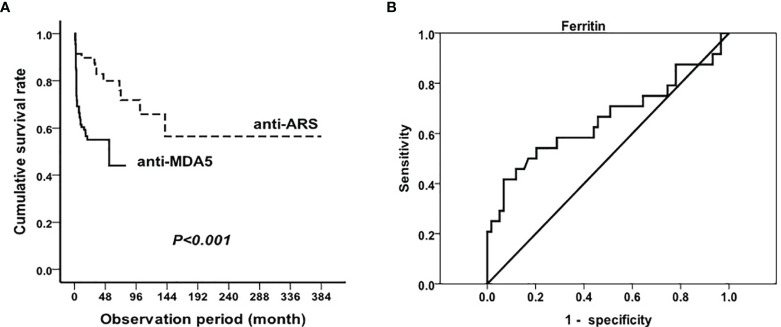
Mortality in RP-ILD in anti-MDA5 and anti-ARS positive DM and prognostic value of serum ferritin Survival analysis in RP-ILD patients with anti-MDA5 or anti-ARS antibodies **(A)** and prognostic value of serum ferritin in MDA5^+^DM **(B)**.

The duration of the follow-up period ranged from 1 to 384 months for all patients. The median follow-up time was 32.4 months. Overall, MDA5^+^DM patients with RP-ILD had significantly lower survival rates than in ASS patients (p<0.001, **Figure 3A**). The 1-year survival rates of MDA5^+^DM patients with RP-ILD and ASS patients with RP-ILD were 53% and 89%, respectively. The 5-year survival rates of MDA5^+^DM patients with RP-ILD and ASS patients with RP-ILD were 45% and 76%, respectively. These results indicated MDA5^+^DM patients with RP-ILD had the highest mortality within the first year after disease onset.

Considering serum ferritin as a significant risk factor for RP-ILD in MDA5^+^DM patients, we next explored the optimal cut-off levels of serum ferritin in predicting the mortality of MDA5^+^DM patients. Of the 110 anti-MDA5 antibody-positive patients with RP-ILD, 48 died during follow-up due to exacerbation of ILD or infection. A total of 34 (70.8%) patients died within 6 months from disease onset, and 43 (89.6%) died within 1 year. Because some of the surviving patients had not been followed up for >1 year, we used ROC analysis to study the value of ferritin required to predict patient’s mortality within half a year. As shown in **Figure 3B**, the optimal cutoff point for serum ferritin was 2200 ng/mL (AUC, 0.66 (95% CI 0.51–0.80). Using the manufacturer’s cutoff value for serum ferritin ≥ 2200 ng/mL, the prognostic sensitivity was 41.7% (95% CI 20.4%–62.9%), whereas the prognostic specificity was 93.2% (95% CI, 86.6%–99.8%). In addition, the positive predictive value for serum ferritin was 71.4% (95% CI 44.4%–98.5%), and the negative predictive value was 79.7% (95% CI 70.0%–89.4%).

## Discussion

The present study indicated the following: (1) MDA5^+^DM was related to a higher frequency of RP-ILD and a worse prognosis than ASS; (2) There were great different multivariable risk factors for RP-ILD in MDA5^+^DM and ASS. A more unique result showed serum hyper-ferritin was the risk factor and prognostic value for RP-ILD in MDA5^+^DM patients but not in ASS patients.

Previous studies had demonstrated that MDA5^+^DM associated ILD can frequently progress rapidly and have a poor outcome, whereas ASS associated ILD usually responds to therapy. The comparison of the incidence of RP-ILD between the two groups was consistent with observations from literature reports; however, in our cohort, ASS patients could also have RP-ILD, especially in patients with anti-PL-7; however, no statistical difference in incidence compared with that in other subgroups of anti-ARS antibodies was observed.

We further selected patients with RP-ILD and compared the clinical differences between the two groups of patients with different antibodies. MDA5^+^DM patients were more likely to have typical dermatomyositis rashes, whereas more ASS patients have elevated CK levels. Considering that RNA helicase encoded by MDA5 is a key molecule participating in the innate immune defense against viruses, viral infection may play an important role in the pathogenesis of DM with anti-MDA5 antibody positivity and RP-ILD (13). In our study, MDA5^+^DM patients with RP-ILD were more likely to have elevated transaminase and ferritin levels and decreased lymphocyte counts, which is consistent with the clinical characteristics of viral infection, and also reflects the difference in pathogenesis of the two groups of patients with RP-ILD having different antibodies.

In addition to the differences in clinical indicators, we also analyzed the survival of the two groups of patients. To our best knowledge, this is the first large sample study showing the difference in survival of patients with RP-ILD having either anti-MDA5 or anti-ARS antibodies. A study on the survival of MDA5^+^DM patients conducted by Koga T et al. suggested that all of the deaths of MDA5^+^DM patients occurred within the first six months from the first visit to the hospital (14). Another study on the prognostic significance of ASS patients indicated that the 10-year survival rate was also significantly higher in the ASS group than in the non- ASS group (15). Our research found that patients with RP-ILD having anti-MDA5 antibody had significantly lower survival rates than those having anti-ARS antibody. Furthermore patients with anti-MDA5 antibody had the highest mortality within the first year of disease onset. Thus, for patients with RP-ILD having anti-MDA5 antibody, early and more powerful treatment is critical.

Anti-MDA5 antibody- and anti-ARS antibody-positive patients have some similarities in terms of the risk factors of RP-ILD. Elevated tumor markers indicated an increased risk of RP-ILD in both groups, consistent with our previous observations (5); however, the exact function of tumor-associated markers in IIM-ILD remains unknown. In the present study, arthralgia was identified as a protective factor for the development of RP-ILD in both groups, which was different from the observations of other research teams (16). This may be owing to differences in sample sizes; therefore, further confirmation studies are needed.

Furthermore, for MDA5^+^DM patients, we found periphery CD4^+^ and CD8^+^ T cells in the RP-ILD group, were significantly lower than in chronic ILD group, confirming previous clinical findings (6, 17), and indicating that T cells play an important role in the development of disease. Knowledge on exactly how the lymphocytes, especially the T cells, work in MDA5^+^DM patients requires more research. Previous research has speculated that lymphocytes migrate to the lungs to take part in the local immune response, causing a decrease of periphery blood lymphocytes (17). Because the onset of MDA5^+^DM may be associated with a history of pre-infection, we speculate that infection by viruses or other pathogens also causes the depletion of lymphocytes in MDA5^+^DM patients. In addition to abnormal T cells, an elevated level of B2 (CD5^-^CD19^+^) cells was also a risk factor for RP-ILD in the MDA5^+^DM group. Rituximab is effective in some MDA5^+^DM patients with RP-ILD (18). Our results and subsequent further research should provide a trial basis for the application of clinical drugs.

Studies have suggested that serological markers are associated with the prognosis of MDA5^+^DM patients, including CRP and KL-6 (3). Nevertheless, our study with a larger sample size showed that serum ferritin was an independent risk factor for poor prognosis, thus providing more parameters for evaluating the prognosis of MDA5^+^DM patients, especially those with RP-ILD. Hyperferritinemia has been considered a predictor of poor outcomes for patients with DM having anti-MDA5 antibody; however different researches have different cutoff values of ferritin for prognosis. The research by Gono et al. showed that all patients with a ferritin level of >1600 ng/mL died of respiratory failure (8) while Kurasawa et al. reported that a ferritin level of >1000 ng/mL before treatment was a risk factor for poor outcome in MDA5^+^DM patients (9). Lian et al. established a mortality risk score model in amyopathic dermatomyositis-associated ILD, identifying a ferritin level of 636 ng/mL as the optimal clinical threshold (4). In this study, we aimed to provide a more accurate cutoff value of ferritin for the prognosis of RP-ILD for MDA5^+^DM patients. Through ROC curve analysis, a serum ferritin level of >2200 ng/mL predicted mortality within half a year in MDA5^+^DM patients with RP-ILD with a sensitivity of 41.7% and specificity 93.2%. Based on our previous observations, the pathogenesis of DM may be related to the activation of macrophages (5, 19), and in this study, patients with RP-ILD having anti-MDA5 antibodies tended to have hyperferritinemia and lower NK cell counts, supporting previous observations.

## Conclusion

There were significant different mortality and multivariable risk factors for RP-ILD in MDA5^+^DM patients and ASS patients. Potential clinical benefits of using these different risk factors deserve assessment of severity and prognosis in IIM patients. Further studies are required to elucidate any difference in pathogenesis between anti-MDA5 antibody and anti-ARS antibody positive patients.

## Data Availability Statement

The raw data supporting the conclusions of this article will be made available by the authors, without undue reservation.

## Ethics Statement

The studies involving human participants were reviewed and approved by Human Ethics Board of the China-Japan Friendship Hospital (approval number: 2016-117). The patients/participants provided their written informed consent to participate in this study.

## Author Contributions

YZ, LY, FC, and YS collected and analyzed the data. XL, GW, and XS provided clinical samples and approved the manuscript. All authors contributed to the article and approved the submitted version.

## Funding

This work was supported by the Youth Program of the National Natural Science Foundation of China (No. 81601367 and 81401363), the Fundamental Research Funds for the Central Universities (No. 3332020074), and the Elite Medical Professionals project of China-Japan Friendship Hospital (NO.ZRJY2021-GG14).

## Conflict of Interest

The authors declare that the research was conducted in the absence of any commercial or financial relationships that could be construed as a potential conflict of interest.

## Publisher’s Note

All claims expressed in this article are solely those of the authors and do not necessarily represent those of their affiliated organizations, or those of the publisher, the editors and the reviewers. Any product that may be evaluated in this article, or claim that may be made by its manufacturer, is not guaranteed or endorsed by the publisher.

## Abbreviations

ALT, alanine transaminase; ARS, aminoacyl-tRNA synthetase; ASS, anti-synthetase syndrome; AST, aspartate transaminase; BALF, bronchoalveolar lavage fluid; CK, creatine kinase; DM, dermatomyositis; EJ, glycyl-tRNA synthetase; FEV1, forced expiratory volume in the first second; FVC, forced vital capacity; IIM, idiopathic inflammatory myopathy; ILD, interstitial lung disease; Jo-1, histidyl tRNA synthetase; LDH, lactate dehydrogenase; MDA5, melanoma differentiation-associated protein 5; MSA, myositis-specific antibody; OJ, isoleucyl-tRNA synthetase; PBL, lymphocytes in the peripheral blood; PL-7, threonyl tRNA synthetase; PL-12, alanyl tRNA synthetase; RP-ILD, rapidly progressive interstitial lung disease.

## References

[1] Douglas WW Tazelaar HD Hartman TE Hartman RP Decker PA Schroeder DR. Polymyositis-Dermatomyositis-Associated Interstitial Lung Disease. Am J Respir Crit Care Med (2001) 164(7):1182–5. doi: 10.1164/ajrccm.164.7.2103110 11673206

[2] Won Huh J Soon Kim D Keun Lee C Yoo B Bum Seo J Kitaichi M. Two Distinct Clinical Types of Interstitial Lung Disease Associated With Polymyositis-Dermatomyositis. Respir Med (2007) 101(8):1761–9. doi: 10.1016/j.rmed.2007.02.017 17428649

[3] Gono T Masui K Nishina N Kawaguchi Y Kawakami A Ikeda K. Risk Prediction Modeling Based on a Combination of Initial Serum Biomarkers in Myositis-Associated Interstitial Lung Disease. Arthritis Rheumatol (2021) 73(4):677–86. doi: 10.1002/art.41566 33118321

[4] Lian X Zou J Guo Q Chen S Lu L Wang R. Mortality Risk Prediction in Amyopathic Dermatomyositis Associated With Interstitial Lung Disease: The FLAIR Model. Chest (2020) 158(4):1535–45. doi: 10.1016/j.chest.2020.04.057 32428508

[5] Zuo Y Ye L Liu M Li S Liu W Chen F. Clinical Significance of Radiological Patterns of HRCT and Their Association With Macrophage Activation in Dermatomyositis. Rheumatol (Oxf) (2020) 59(10):2829–37. doi: 10.1093/rheumatology/keaa034 32065646

[6] Chen F Wang D Shu X Nakashima R Wang G. Anti-MDA5 Antibody Is Associated With A/SIP and Decreased T Cells in Peripheral Blood and Predicts Poor Prognosis of ILD in Chinese Patients With Dermatomyositis. Rheumatol Int (2012) 32(12):3909–15. doi: 10.1007/s00296-011-2323-y 22198664

[7] Shi J Li S Yang H Zhang Y Peng Q Lu X. Clinical Profiles and Prognosis of Patients With Distinct Antisynthetase Autoantibodies. J Rheumatol (2017) 44(7):1051–7. doi: 10.3899/jrheum.161480 28461650

[8] Gono T Kawaguchi Y Satoh T Kuwana M Katsumata Y Takagi K. Clinical Manifestation and Prognostic Factor in Anti-Melanoma Differentiation-Associated Gene 5 Antibody-Associated Interstitial Lung Disease as a Complication of Dermatomyositis. Rheumatol (Oxf) (2010) 49(9):1713–9. doi: 10.1093/rheumatology/keq149 20498012

[9] Kurasawa K Arai S Namiki Y Tanaka A Takamura Y Owada T. Tofacitinib for Refractory Interstitial Lung Diseases in Anti-Melanoma Differentiation-Associated 5 Gene Antibody-Positive Dermatomyositis. Rheumatol (Oxf) (2018) 57(12):2114–9. doi: 10.1093/rheumatology/key188 30060040

[10] Bohan A Peter JB. Polymyositis and Dermatomyositis (First of Two Parts). N Engl J Med (1975) 292(7):344–7. doi: 10.1056/NEJM197502132920706 1090839

[11] Mammen AL Allenbach Y Stenzel W Benveniste O E.t.W.S. Group. 239th ENMC International Workshop: Classification of Dermatomyositis, Amsterdam, the Netherlands, 14-16 December 2018. Neuromuscul Disord (2020) 30(1):70–92. doi: 10.1016/j.nmd.2019.10.005 31791867

[12] Cavagna L Trallero-Araguas E Meloni F Cavazzana I Rojas-Serrano J Feist E. Influence of Antisynthetase Antibodies Specificities on Antisynthetase Syndrome Clinical Spectrum Time Course. J Clin Med (2019) 8(11):2013. doi: 10.3390/jcm8112013 PMC6912490 31752231

[13] Sato S Hoshino K Satoh T Fujita T Kawakami Y Fujita T. RNA Helicase Encoded by Melanoma Differentiation-Associated Gene 5 Is a Major Autoantigen in Patients With Clinically Amyopathic Dermatomyositis: Association With Rapidly Progressive Interstitial Lung Disease. Arthritis Rheum (2009) 60(7):2193–200. doi: 10.1002/art.24621 19565506

[14] Koga T Fujikawa K Horai Y Okada A Kawashiri SY Iwamoto N. The Diagnostic Utility of Anti-Melanoma Differentiation-Associated Gene 5 Antibody Testing for Predicting the Prognosis of Japanese Patients With DM. Rheumatol (Oxf) (2012) 51(7):1278–84. doi: 10.1093/rheumatology/ker518 22378718

[15] Hozumi H Enomoto N Kono M Fujisawa T Inui N Nakamura Y. Prognostic Significance of Anti-Aminoacyl-tRNA Synthetase Antibodies in Polymyositis/Dermatomyositis-Associated Interstitial Lung Disease: A Retrospective Case Control Study. PLoS One (2015) 10(3):e0120313. doi: 10.1371/journal.pone.0120313 25789468 PMC4366175

[16] Hoa S Troyanov Y Fritzler MJ Targoff IN Chartrand S Mansour AM. Describing and Expanding the Clinical Phenotype of Anti-MDA5-Associated Rapidly Progressive Interstitial Lung Disease: Case Series of Nine Canadian Patients and Literature Review. Scand J Rheumatol (2018) 47(3):210–24. doi: 10.1080/03009742.2017.1334814 29065773

[17] Huang W Ren F Luo L Zhou J Huang D Pan Z. The Characteristics of Lymphocytes in Patients Positive for Anti-MDA5 Antibodies in Interstitial Lung Disease. Rheumatol (Oxf) (2020) 59(12):3886–91. doi: 10.1093/rheumatology/keaa266 32535634

[18] Ogawa Y Kishida D Shimojima Y Hayashi K Sekijima Y. Effective Administration of Rituximab in Anti-MDA5 Antibody-Positive Dermatomyositis With Rapidly Progressive Interstitial Lung Disease and Refractory Cutaneous Involvement: A Case Report and Literature Review. Case Rep Rheumatol (2017) 2017:5386797. doi: 10.1155/2017/5386797 29225988 PMC5684540

[19] Peng QL Zhang YL Shu XM Yang HB Zhang L Chen F. Elevated Serum Levels of Soluble CD163 in Polymyositis and Dermatomyositis: Associated With Macrophage Infiltration in Muscle Tissue. J Rheumatol (2015) 42(6):979–87. doi: 10.3899/jrheum.141307 25877505

